# Translational Functional Neuroimaging in the Explanation of Depression

**DOI:** 10.4274/balkanmedj.2017.1160

**Published:** 2017-12-01

**Authors:** Drozdstoy Stoyanov, Sevdalina Kandilarova, Stefan Borgwardt

**Affiliations:** 1 Department of Psychiatry and Medical Psychology, Medical University of Plovdiv, Plovdiv, Bulgaria; 2 Research Complex for Translational Neuroscience, Medical University of Plovdiv, Plovdiv, Bulgaria; 3 Department of Psychiatry, University of Basel, Basel, Switzerland

**Keywords:** Neuroscience, translational medical research, depression, neuroimaging, functional

## Abstract

Translation as a notion and procedure is deeply embodied in medical science and education. Translation includes the possibility to translate data across disciplines to improve diagnosis and treatment procedures. The evidence accumulated using translation serves as a vehicle for reification of medical diagnoses. There are promising, established post hoc correlations between the different types of clinical tools (interviews and inventories) and neuroscience. The various measures represent statistical correlations that must now be integrated into diagnostic standards and procedures but this, as a whole, is a step forward towards a better understanding of the mechanisms underlying psychopathology in general and depression in particular. Here, we focus on functional magnetic resonance imaging studies using a trans-disciplinary approach and attempt to establish bridges between the different fields. We will selectively highlight research areas such as imaging genetics, imaging immunology and multimodal imaging, as related to the diagnosis and management of depression.

Depression has a high social burden and affects over 350 million people worldwide. According to the World Health Organization (WHO), by 2030, depression is expected to be the second most common disease worldwide (1). The symptoms of depression are multifaceted and include low mood; anxiety; lack of pleasure and interest; decreased self-esteem; thoughts of guilt, hopelessness, and even suicide; disturbances of sleep and appetite; and low concentration and various somatic complaints. Despite recent advances in molecular, genetic and imaging research, the exact pathophysiological mechanisms underlying depression remain elusive, and its treatment remains far from being successful; just 11% of the patients remit following the first year of antidepressant treatment (2). One of the major reasons for this, we believe, is the lack of reliable biomarkers for the diagnosis of depression, as well as for other psychiatric disorders.

## Data translation and reification of diagnosis

Translation as a notion and procedure is deeply embodied in medical science and education. Translation includes the possibility to translate data across disciplines and across domains within a single discipline to improve diagnosis and treatment procedures. The evidence accumulated using translation serves as a vehicle for reification of medical diagnoses. Reification and hypostatization in the sense of G. Lucacs refer to objectification (here, with biological evidence) of constructs which are otherwise a matter of temporary social conventions (3). Below we attempt to dissect the status of translation and reification of diagnosis in medicine as compared to psychiatry.

## The typical case: Myocardial infarction

Myocardial infarction as clinical nosology entails detailed translation across four bottom-up disciplinary domains (biochemistry, physiology, radiology and clinical evaluation) (4):

**Biochemistry:** The hypostasis of infarction is reflected in biochemical mechanisms of hypoxia and ischemia, which results in necrosis. Damaged cells release creatinin-phosphokinase MB and troponin into the circulation. These substances are elevated in the acute phase of infarction (with up to 78% specificity), and they are considered biomarkers of disease. However, it should be emphasized that those biomarkers themselves do not belong to the realm of clinical cardiology per se, but are rather translated for use in clinical cardiology methods and data from biochemistry. Without translation to clinical symptoms and observations, they are meaningless and might be regarded as indirect measures of various processes in the living organism. To put it in the framework of reification, it is troponin that reifies the diagnosis, and it is the clinical hypothesis that reifies the troponin as a biomarker.

**Physiology:** The other basic domain involved in this exemplary case is electrophysiology. On the electrocardiogram (ECG) record, a deviation is observed, known as elevation of the ST segment. Once again, taken as a sole measure, it has limited sense. It requires translation to interpret it as a marker of infarction in the context of the patient’s complaints. Furthermore, it needs to be translated to the other measures as discussed here.

**Radiology:** Radiological scans include the x-ray contrast - the based technique of coronary angiography. This provides real-time images of where and to what extent are the coronary vessels are obstructed. In this sense, angiography reflects on the primary cause of infarction: the obstructed blood and oxygen supply. Radiology is not cardiology from an epistemological point of view. Yet the data acquired inside this domain are successfully translated to reify the diagnosis.

Thus, a circular process of translation is implemented: radiography gives information about the effects of the cause, biochemistry and electrophysiology provide correlates of the mechanisms that cause this disorder, and (taken together) these measures reify the diagnosis, which in turn motivates therapeutic intervention.

**FIG. 1.**
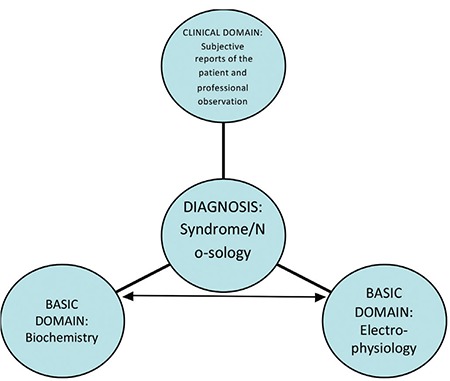
Translation and triangulation of data in biomedicine.

In the common case, not all medical settings have at their disposal a clinical laboratory or angiography facilities. For this reason, minimum resource methods are often use, such as ECG to perform the diagnostic assessment. What makes such an approach feasible is the triangulation of knowledge as acquired within discrete disciplinary matrices (Fig. 1). Under the triangulation model, diagnosis is sustained by a nomothetic network of translational connections to other disciplines, whereby it is reified in terms of biological measures, which penetrate into the substrate of disease. This allows the medical diagnosis data to maintain the stability that facilitates the process of translation and extrapolation from statistical units (average group data) to individual, day-to-day clinical decision-making. Due to the immense heterogeneity and variability of the reported data in neuropsychiatry, this process is much more complicated and does not allow us to transfer knowledge from research to clinical utility, which is demonstrated in the next case study (5).

## Atypical case: Depression

Translation and reification in psychiatry are still out of reach. All data acquired externally to the domain of clinical psychopathology are regarded as irrelevant to diagnosis. This refers to most recent and current studies in basic neuroscience, which are supposed to be involved in translation [e.g., genetics, immunology, electroencephalography (EEG), and functional neuroimaging (e.g., focus on functional magnetic resonance imaging, fMRI)] (6). In reality, triangulation is performed within one and the same realm of knowledge, and it is the realm of narrative, either professional (interviews) or patient’s (self-assessment), whereby both converge into the quantitative measures, named scales. Scales are usually composed of structured questions/statements, called items, which require a different kind of item response.

Although psychological and psychiatric assessment tools (scales) belong nominally to different disciplinary frameworks, they share the same matrix: the matrix of statistically quantified subjective and inter-subjective narratives.

If we assume the case of depression, then the translation across disciplines to operationalize diagnosis will appear like this:

Psychiatry and clinical psychology use four levels of clinical tools (free clinical interview, structured or semi-structured interviews, clinician-rated scales and self-assessment scales). Psychiatry more often operates with structured clinician-rated scales, while clinical psychology refers more frequently to self-evaluation instruments. The most common among the psychiatric tools in depression assessment are the Montgomery-Asberg Depression Rating Scale (MADRS) and Hamilton Scale for Depression (HAM-D). These consist of items rated according to a Likert scale, the score of which is taken to have diagnostic value. For instance, a score on MADRS over 20 is considered as a measure of mild or moderate depression and a score ranging above 30 to be an indicator of severe depression. The usual psychological tests (or inventories) that are implicated in depression are Beck’s and Zung’s inventories or Depression Scale by Von Zerssen (e.g., a score of ~20 points on Von Zerssen DS is supposed to correspond to the >20 MADRS evaluation and is, therefore, taken to support the diagnostic hypothesis).

**FIG. 2.**
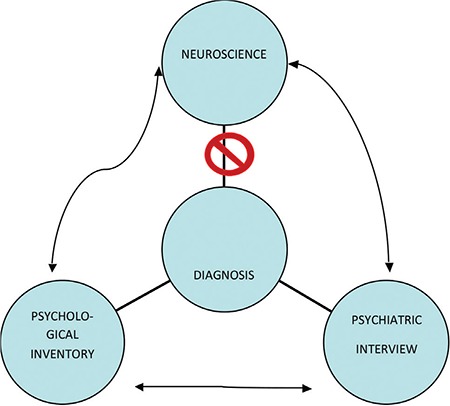
Translation and triangulation of data in psychiatry.

There is no agent of reification located in the realm of objective biological facts (bio-marker) in this framework, and thus much confusion is caused by diagnostic errors, overestimating or underestimating the clinical data (Figure 2). Any discrepancy/dissociation between the two measurement scores is interpreted as a rule in favour of the observer-based evaluation (the interview). Still, there are no common guidelines to regulate this.

However, there are established promising post hoc correlations between the different types of clinical tools (interviews and inventories) and neuroscience. Thus, the different kinds of measures represent statistical correlations that need to be integrated into diagnostic standards and procedures but this, as a whole, is a step forward towards a better understanding of the mechanisms underlying psychopathology in general and depression in particular. Below, we will focus on fMRI studies using a trans-disciplinary approach and attempt to establish bridges between the different fields. We will selectively highlight research areas such as imaging genetics, imaging immunology and multimodal imaging, as related to the diagnosis and management of depression.

## Genetic neuroimaging

Imaging genetics is a rapidly developing field in which neuroimaging is used to elucidate variations in brain structure and function as they relate to genotype (7). Classical genetic studies of depression have revealed associations with genes for serotonin and dopamine receptors and transporters (8,9) but these are not specific to depression, being found in psychiatric diagnosis such as anxiety disorders, obsessive-compulsive disorder, bipolar disorder, and schizophrenia (10). Amongst the most studied is a polymorphism in the promoter region (5-HTTLPR) of the serotonin transporter gene (SLC6A4) commonly reported with two variants: the l (long) allele, linked to increased concentrations of 5-HTT messenger RNA and increased serotonin (5-HT) uptake, and the s (short) allele, associated with decreased 5-HT uptake (11).

The s-allele has been related to negative emotions and increased amygdala activation during perception of aversive emotional stimuli, which is also characteristic for depression (12). Association was found between the s allele and greater functional connectivity (FC) between the amygdala and anterior medial prefrontal cortex (mPFC) (13). While replicating these findings, Pezawas et al. (14) also reported reduced FC between the amygdala and medial prefrontal regions located to the pre- and sub-genual anterior cingulate cortex (ACC). These findings suggest that the s allelic variation of the serotonin transporter gene has a specific influence on the fronto-limbic circuit, up-regulating connectivity between the amygdala and anterior mPFC and down-regulating FC between the amygdala and perigenual ACC.

The dysfunction of the fronto-limbic circuit is a well-replicated finding in depression (15), and the risk of developing the disorder is associated with 5-HTTLPR polymorphism (16). Thus, it is possible to suggest that the serotonin transporter gene’s involvement in the development of depression is mediated by its influence on the function of the limbic system. In accordance with this, recent fMRI studies in depressed patients have shown that carriers of at least one s-allele have increased amygdala activation, both at rest and while viewing emotionally valenced stimuli (17,18,19).

Also, the activation of the prefrontal cortex, as well as its connectivity with the amygdala, were different in depressed patients with low- and high-expression genotype (20). According to the authors, the increased medial prefrontal activation and mPFC-amygdala connectivity may counteract the risk for major depressive disorder (MDD) in healthy carriers of 5-HTT low-expression alleles, while this protective factor might be lost in patients who suffer from MDD.

Earlier genetic studies have also addressed the question of the influence of the SCL6A4 variants on treatment response in depressed patients and have found that s-allele carriers had poorer response to antidepressants, such as escitalopram (21), and more adverse effects to paroxetine but less to mirtazapine (22); although more recent study found no influence on sertraline efficacy among different ethnic groups (23). Some large clinical studies (24) and meta-analyses failed to prove or found only a modest effect of the 5-HTT genotype on treatment response (25,26).

The catechol-O-methyltransferase (COMT) gene with its val158met polymorphism have been extensively studied in psychiatric genetics (mainly in relation to schizophrenia). Met-homozygotes have been associated with the lower enzymatic activity of COMT compared to val-homozygotes (27), with consequently higher cortical dopamine concentrations (28). In a large multi-centred study, an association was found between the high-activity COMT Val allele, particularly the COMT Val/Val genotype, and early onset depression (29). A recent meta-analysis, on the other hand, failed to find such a link (30).

fMRI research has explored the influence of the COMT genotype on both emotional processing and working memory in major depression (31). In healthy controls, a positive correlation was found between the number of met-alleles and right inferior frontal gyrus activation during emotional processing, that was not present in depressed subjects. Moreover, there was an effect of genotype in a cluster including the amygdala and hippocampus. No differential effect of genotype was found under the working memory task as in both groups carrying the met-allele was associated with lower activation in the middle frontal gyrus.

In bipolar patients, implicit processing of fearful and angry faces elicited significant positive connectivity between the amygdala, dorsolateral prefrontal cortex (DLPFC) and supramarginal gyrus in Val/Val carriers and significant negative connectivity between DLPFC and AMG in Met carriers (32). Interestingly the antidepressant effect of SSRIs (fluoxetine and paroxetine) was found to be worse in Val/Val homozygotes, which might be reflecting the supposed dysfunction of the top-down dopaminergic regulation in the frontolimbic circuit (33,34).

Another widely studied genotype in depression is the Val66Met polymorphism of the gene for the brain-derived neurotrophic factor (BDNF) that plays a major role in neuroplasticity (35). In healthy controls, the met-allele was found to negatively influence hippocampal activity during a declarative memory task and positively influence amygdala activity in an emotional processing task (36,37). Similarly, while investigating a sample of adolescents suffering from depression and anxiety, researchers found that Met-carriers showed greater amygdala responses to emotional faces than Val/Val homozygotes (38).

Serum BDNF-levels are consistently found to be lower in depressed patients and to normalize following treatment with a variety of antidepressants, including venlafaxine, sertraline, fluoxetine, paroxetine, lithium and citalopram, as well as with vagus nerve stimulation, repetitive transcranial magnetic stimulation and electroconvulsive therapy (39,40,41,42,43). In relation to treatment outcome, the Met-allele carriers demonstrated a better response to treatment with citalopram in MDD (44). However, a meta-analysis suggested that there is no association between this BDNF polymorphism and hippocampal volumes. For each BDNF genotype, the hippocampal volumes were significantly lower in neuropsychiatric patients (including depression) than in healthy controls (45).

Recently, the interaction between single genetic variants became a focus of research, providing valuable insight into the pathophysiological mechanisms of psychopathology. In healthy subjects, an additive effect of COMT and 5-HTT risk variants polymorphisms (met and s - respectively) was observed, accounting for 40% of the inter-individual variance in the averaged blood-oxygen-level-dependent (BOLD) response of amygdala, hippocampal and limbic cortical regions elicited by unpleasant stimuli (46). In a fMRI study with an oddball emotional paradigm, the participants with both the s allele of the 5-HTTLPR and met allele of the BDNF gene exhibited increased activation to sad stimuli in the subgenual cingulate and posterior cingulate (47).

Apart from gene × gene interactions, gene × environment interactions have recently attracted increasing scientific attention. Amongst the most prominent is the interaction between stressful life events and the serotonin genotype in the development of depression (48). Individuals with one or two copies of the s allele of the SCL6A4 gene demonstrated more depressive symptoms, diagnosable depression, and suicidality in relation to stressful life events than one allele homozygotes (49). Also, amygdala activation to face stimuli correlated negatively with stressful life events (SLEs) in the S-group and correlated positively with life stress in the L-group of healthy subjects (48,50). A later study found that this interaction was accounted for by major interpersonal SLEs but not by major non-interpersonal SLEs (51).

The above findings support existing hypothesis of depression as a polygenic multifactorial disorder (in light of the biopsychosocial model) that is the endpoint of diverse combinations of genetic susceptibility (5-HTT, COMT, BDNF genes) and environmental influences (stress), which converge into dysfunctional neurotransmitter systems and neurocircuits. Stress has been associated with the development of depression, as well as other psychiatric and somatic disorders, most probably through its influence on the immune system (52).

## Psychoneuroimmunology and imaging

There is growing evidence of increased inflammatory biomarkers in depressed patients, including elevation of acute-phase proteins and the production of inflammatory cytokines, as well as increased cellular markers of immune activation (53). It is important to note that apart from differences in mean levels of inflammatory markers between depressed patients and controls, significant associations between blood concentrations of inflammatory factors and the severity of depressive symptoms, including cognitive dysfunction, fatigue and impaired sleep, have also been reported (54).

Another major body of data in support of inflammation’s role in depression comes from the findings that administration of inflammatory cytokines or cytokine inducers can cause a depressive-like behaviour in both laboratory animals and humans. Also, treatment with interferon-alfa results in a clinically significant depression in up to 40% of the cases (55). Several pathways have been proposed for the explanation of the involvement of cytokines in the pathophysiology of depression, including alteration of neurotransmitter metabolism and neurocircuitry (56), neurogenesis (57), and neuroendocrine function (58).

The neurotransmitters serotonin, dopamine, and glutamate all seem to be affected by cytokines. The serotonin system, for instance, might be influenced by metabolic changes [e.g., increased activity of the indoleamine 2,3-dioxygenase, leading to decreased concentration of the precursor tryptophan, and increased levels of kynurenine metabolites with toxic effects on the brain (59)], as well as through the activity of the serotonin transporter [which is increased after acute administration of cytokines and in depressed patients (60,61)].

Similarly, the metabolism of dopamine seems to be affected by cytokines, as evidenced by a recent study in patients treated with interferon (IFN) alpha, where increased IL-6 in the cerebro-spinal fluid (CSF) was associated with decreased CSF tetrahydrobiopterin (BH4) - an essential enzyme co-factor for tryptophan hydroxylase and tyrosine hydroxylase, which are the rate-limiting enzymes for the synthesis of serotonin and dopamine, respectively. In this study, significant correlations were found between the phenylalanine (Phe) to tyrosine (Tyr) ratio and CSF concentrations of both dopamine and its metabolite, homovanillic acid (62).

The Phe/Tyr ratio has been used as an indirect measure of BH4 activity in the brain and has been correlated with symptoms of depression in elderly individuals (63).

Following IFN-alpha treatment, patients with hepatitis C demonstrated significantly decreased activation of ventral striatal regions during a hedonic reward task, which in turn was highly correlated with anhedonia, depression, and fatigue (64). A complementing positron emission tomography (PET) study found significantly increased 18F-dopa uptake and decreased 18F-dopa turnover in caudate and putamen and the same ventral striatal regions identified in the fMRI. Interestingly, reduced 18F-dopa uptake at baseline was predictive of interferon alfa-induced behavioural changes, indicating that dopaminergic tone may also serve as an important vulnerability factor regarding the impact of cytokines on behaviour. Similar changes in ventral striatal activity were observed in a monetary reward task after administration of endotoxin (65).

While exploring inflammation induced mood change in healthy controls with fMRI, Harrison et al. (66) found that the significant deterioration in total mood following injection of typhoid vaccine was highly correlated with enhancement of evoked responses to emotional facial expressions within the subgenual ACC, which has been implicated in the pathophysiology of depression. Moreover, inflammation-associated change in total mood correlated with a reduction in connectivity between subgenual cingulate and bilateral nucleus accumbens, which is a dopamine-regulated reward-related region that is implicated in anhedonia. Interestingly, in females (but not males) exposed to endotoxin, increases in IL-6 were associated with increases in social pain-related neural activity (dorsal anterior cingulate cortex, anterior insula) that mediated the relationship between IL-6 increases and depressed mood increases (67).

A recent magnetic resonance spectroscopy (MRS) study on unmedicated MDD patients revealed an interesting positive correlation between C-reactive protein (CRP) levels and the concentration of glutamate in the basal ganglia, which was associated with anhedonia and psychomotor slowing (68). In a sample of depressed patients, plasma CRP levels were found to be negatively associated with functional connectivity between the left inferior ventral striatum and ventromedial prefrontal cortex (vmPFC). In the same sample, anhedonia and psychomotor slowing were also negatively correlated with connectivity between striatal and vmPFC regions that are considered to be part of the reward circuit (69).

Investigation of patients with IFN-induced depression has provided valuable insight into inflammation’s effects on neurogenesis and neurodevelopment. BDNF levels were reduced following treatment with IFN-alpha, and lower pretreatment BDNF was associated with higher depression symptoms (70). In animal models, peripheral immune activation, induced by injection of lipopolysaccharide, was followed by decreased BDNF in the hippocampus (-20%), frontal cortex (-19%), parietal cortex (-63%), temporal cortex (-29%) and occipital cortex (-41%), as well as decreased nerve growth factor and Neurotrophin-3 (71).

Changes in the activity of hypothalamus-pituitary axis (HPA) has been found both in stress and depression (72). Also, several lines of research have supported the notion of significant effects of cytokines on the HPA. Both in vitro and in vivo studies have demonstrated that inflammatory factors change the activity of the HPA by influencing the release of the corticotropin-releasing hormone by the paraventricular nucleus, the release of adrenocorticotropic hormone (ACTH) by the pituitary gland and of cortisol by the adrenal glands (73). Interestingly, exaggerated ACTH and cortisol response to the initial injection of IFN-alpha predicted the subsequent development of IFN-alpha-induced depression in malignant melanoma patients (74).

The activity of the HPA has been linked to the functional changes in brain areas involved in emotion processing in bipolar and MDD patients, as well as healthy controls during a facial emotion perception paradigm. In both groups, cortisol was associated with greater activation in several regions involved in the perception and control of emotion (dorsal anterior cingulate, inferior parietal lobule, insula, putamen, precuneus, middle and medial frontal and postcentral gyri, posterior cingulate, and inferior temporal gyrus during emotion processing of all faces). However, in the patient group, cortisol responsivity was associated with hypoactivation of the insula, postcentral gyrus, precuneus, and putamen for fearful faces and the medial frontal gyrus for angry faces (75).

The findings highlighted so far reveal some of the more specific mechanisms of the pathophysiology of depression and its interplay with stress and inflammation, which again is influencing the same neurotransmitter systems and neurocircuits, as well as neurogenesis and neuroplasticity, that are affected by the genetic predisposition. Below, we will move from the molecular level to the systems levels, as reflected by electrophysiology and multimodal functional imaging.

## Multimodal imaging

## Functional MRI and EEG

Combined EEG-fMRI investigates neural activity, providing both high spatial and high temporal resolution specific to each of the techniques separately (76,77). Moreover, this method enables the translation of knowledge from the vast field of electrophysiology to the rapidly growing field of functional neuroimaging and vice versa. Most of the studies using this technique have focused on areas such as emotion processing and regulation, reward processing, and working memory in healthy populations (78,79,80,81). Although research in depressed patients remains sparse, some of the relevant studies in non-clinical samples will be reviewed below.

Simultaneous EEG-fMRI acquisition during working memory (WM) task revealed a functional relation between WM-induced right posterior alpha increases and decreases in BOLD activity of the right middle temporal gyrus and primary visual cortex. The WM-induced frontal theta power, on the other hand, was associated with decreased activity in a set of regions that together form the default mode network (DMN) (82). The increased activity of the DMN in depressed patients is a well-replicated finding supposedly reflecting heightened self-referential processes (83).

The activity of the DMN was found to be positively correlated with visual alpha power in the eyes-open condition but not in the eyes-closed condition, which is the most common in resting state fMRI (84). This last study might have resolved previous inconsistencies in the reported relation between EEG alpha and default mode network activity. Our research suggests that antidepressant response is predicted by high alpha power in MDD patients treated with various antidepressants (85).

About emotional picture processing, a combined EEG-fMRI approach revealed interesting valence-specific findings. During the pleasant condition, the late positive potential (LPP - a reliable electrophysiological measure of emotional perception) was correlated with BOLD in the occipitotemporal junction, medial prefrontal cortex, amygdala, and precuneus. For the unpleasant condition, the significant LPP-BOLD correlation was found in the ventrolateral prefrontal cortex, insula, and posterior cingulate cortex (86). In a seed-based connectivity study on this same dataset, FC of mPFC and amygdala with a plethora of other cortical regions was found to be stronger in trials with larger LPP (87).

The neural correlates of anhedonia have been studied using EEG, as well as structural and fMRI, during execution of monetary reward task in healthy individuals (88). A negative association was found between anhedonia and nucleus accumbens (NAcc) responses to reward feedback and NAcc volume, whereas anhedonia was positively associated with resting EEG delta activity in rostral ACC.

Exploring a novel real-time fMRI-neurofeedback (rtfMRI-nf) procedure for the treatment of depression, the authors of this recent study implemented simultaneous EEG recordings to reveal objective measures of its efficacy (89). A positive correlation between the changes in frontal EEG alpha asymmetry (FEA) and amygdala BOLD laterally was found in the experimental group compared to the control group and, interestingly, changes in FEA during the rtfMRI-nf task showed a significant positive correlation with the severity of depression. This last finding is consistent with the inverse correlation between depression severity and frontal EEG asymmetry at rest (90).

## Functional MRI and MRS

An increasing amount of data supports the utility of combined fMRI-MRS techniques that could reveal the molecular underpinnings of observed BOLD activity changes in both resting state and task-related functional imaging. In this way, for instance, the concentration of GABA was found to be correlated with the negative BOLD activity of the ACC during emotional processing (91). Moreover, in a task involving cognitive control, low resting-state glutamate levels in the dorsal ACC were associated with an increased BOLD response in the condition with high task demands, whereas high-glutamate levels correlated with an increased BOLD response in low demands tasks (92).

In healthy subjects, BOLD signal changes during empathy task in the supragenual ACC (task-positive region) were related to the level of glutamate in the perigenual ACC (task-negative region). Moreover, there was no opposite relationship between activity in the pgACC and the level of glutamate in the sgACC (93). MDD patients with high anhedonia scores, compared to healthy controls and patients with low anhedonia scores, exhibited significantly lower (glutamine) Gln concentrations in pgACC, and no differences in glutamate (Glu), GABA or n-methyl aspartate (NAA) in the same region (94). In MDD patients, Glu and NAA concentrations in the pgACC correlated with negative BOLD responses (NBRs) to emotional stimulation, whereas in healthy controls, NBRs correlated with GABA instead.

A recent multimodal imaging study combining resting-state fMRI, aversion task fMRI, glutamate MRS, and diffusion MRI explored the impact of negative childhood events [as measured by childhood trauma questionnaire (CTQ) on brain activity in healthy subjects] (95). Higher CTQ scores correlated negatively with mPFC resting state glutamate levels and positively with mPFC entropy. Of note, the mPFC is a key node in both the DMN and emotion affective processing networks, and childhood trauma is a well-established risk factor for depression.

As evidenced by the findings reviewed, the application of combined EEG-fMRI and MRS-fMRI provides a valuable insight into the intimate pathophysiological mechanisms of depression, underscoring the engagement of the same neurotransmitter systems and neurocircuits on both molecular and functional/anatomical levels. However, a critical point in every research in psychiatry is the definition of the diagnostic categories.

## Validation of imaging in the diagnostic process of depression

Compared to healthy controls, depressed patients are characterized by increased activity and resting-state FC between nodes of the DMN (83). However, changes in DMN activity and connectivity are found in other psychiatric conditions, such as schizophrenia and bipolar disorder, which show both common and distinct changes (96,97,98). Similarly, task-related fMRI in depression has revealed changes in the activations of cortical and subcortical structures during emotion processing, cognitive control and working memory that is not specific particularly to MDD but is found in other disorders (99,100,101,102). Moreover, there are huge inconsistencies in the field, supported by a most recent meta-analysis of fMRI and PET studies with cognitive and/or emotional tasks, which failed to reveal any significant results (103).

One of the reasons for these and other inconsistencies might be the fundamental lack of validity of psychiatric diagnoses in contemporary diagnostic classifications (4,104). To overcome this issue, we have conceptualized an approach of translational cross-validation of widely used psychiatric/psychological assessment instruments (e.g., depression scale) with fMRI, thus aiming at bridging the gap between neuroscience and psychiatry (105,106). By implementing this new design, we expected to find significant correlations between the psychological rating scale score and the pattern of BOLD activity. Thus, we can re-validate the clinical assessment tools according to the evidence from the simultaneous cross-validation with the neuroimaging methods.

Recently, we have been able to test our hypothesis on a sample of healthy controls and depressed patients with a new fMRI paradigm, specifically developed for this purpose. We implemented a standard block design where the Diagnostically Specific (DS) blocks consisted of four statements from the von Zerssen Depression Scale (DS), and the DN blocks consisted of four statements from an interest questionnaire. When contrasting the DS with the DN blocks (DS>DN), depressed patients demonstrated residual activations in frontal and central regions, while control subjects had no residual activations. The between-group analysis revealed significant activation in the middle frontal gyrus, parahippocampal gyrus, hippocampus and thalamus in patients compared to controls (107). These initial results are promising and suggest that, in the near future, the clinical assessment tools that we are using in our everyday clinical decisions will be validated by functional neuroimaging.

In this selective review, we have elaborated some of the most recent advances in the field of translational neuroimaging and, in particular, in the translation across genetic imaging, psychoneuroimmunology and multimodal imaging, as well as to the more conceptual issue of reification of diagnosis in psychiatry by means of inter-disciplinary translation. Although these topics appear to be discrete, they have the ultimate potential to further bridge the gap between neuroscience and psychiatry by co-producing a meta-language between disciplines, similar to other fields of medical expertise. We believe that our own approach of translational cross-validation of psychological assessment instruments is just one of many possible solutions to be discovered at the intersections between translational psychiatry and basic neuroscience, thereby transforming it into clinical utility.
